# Psychometric Properties of the Chinese-Language Attitude toward Physical Activity Scale: A Confirmatory Study on Chinese Children

**DOI:** 10.3390/ijerph18179253

**Published:** 2021-09-02

**Authors:** Yanli Zhou, Sensen He, Ke Zhou, Garry Kuan, Ming-Kai Chin, Yee Cheng Kueh, Abdulwali Sabo, Biljana Popeska, J. Larry Durstine

**Affiliations:** 1 School of Physical Education, Henan University, Kaifeng 475001, China; 10180056@vip.henu.edu.cn; 2 Minsheng College, Division of Henan University, Kaifeng 475001, China; 104754170320@vip.henu.edu.cn; 3 Exercise and Sports Science Programme, School of Health Sciences, Universiti Sains Malaysia, Kubang Kerian 16150, Kelantan, Malaysia; 4 The Foundation for Global Community Health, 1550 W Horizon Ridge Pkwy Ste R #206, Henderson, NV 89012, USA; mingkai@gchfoundation.org; 5 Biostatistics and Research Methodology Unit, School of Medical Sciences, Universiti Sains Malaysia, Kubang Kerian 16150, Kelantan, Malaysia; yckueh@usm.my (Y.C.K.); ab.wali@yahoo.co.uk (A.S.); 6 Faculty of Educational Sciences, Goce Delcev Univeristy, 2000 Stip, North Macedonia; biljana.popeska@ugd.edu.mk; 7 Department of Exercise Science, University of South Carolina, Columbia, SC 29208, USA; ldurstin@mailbox.sc.edu

**Keywords:** psychometric, attitude, physical activity, confirmatory, Chinese children

## Abstract

*Background:* This study examined the psychometric properties of the Chinese version of the Attitude toward Physical Activity Scale (APAS) using a cross-sectional design. *Methods:* The sample consisted of 692 primary students in China (boy 52.6%, girl 47.4%). The mean age of the participants was 9.4 years (SD = 0.92). Psychometric properties of the 57-item APAS was examined using confirmatory factor analysis (CFA). *Results:* The hypothesized seven factors model was supported by CFA (CFI = 0.912, TLI = 0.901, SRMR = 0.041, RMSEA = 0.029) after 22 items were removed and the inclusion of seven residual covariance for items loaded on the same factor. Cronbach’s alphas of the scales ranged between 0.50 and 0.76. The composite reliability (CR) was between 0.50 and 0.75. All inter-factor correlation coefficient was less than 0.85. *Conclusions:* Findings provided empirical evidence that the Chinese version of the APAS has adequate psychometric properties for assessing attitudes of primary school children in China toward physical activity.

## 1. Introduction

Over the last few years, regular physical activity has been identified as a crucial factor for healthy lifestyle [1]. People who engaged in regular physical activity were less likely to sustain diabetes (type-2), heart disease, overweight and certain types of cancer [2]. Further, research has also found regular exercise promote formation of neurons in the brain, neuromuscular system maturation, academic performance, as well as reduce rigid posture [3,4,5]. However, international data show that physical inactivity accounted for 3.8% of deaths between 2002 and 2011, making it one of the key risk factors for non-communicable diseases [6,7].

China is a developing country witnessing increasing rates of physical inactivity, despite the documented benefits of regular exercise [6]. Several factors that affect children’s level of physical activity are environments, modern technology, poor eating habit and excessive use of the social network [8,9,10]. These factors lead to physical inactivity in both school and home environments [11]. The social-ecological model [12] highlight the social, natural and built environments as significant factors contributing to physical activity levels of children [1]. These environmental factors surround individual factors such as sex, age, ability, time and motivation [13,14]. As such, the school environment is the appropriate environment to implement physical activity intervention programs because children spent most of their time in schools [15].

An effort to promote and encourage children and adolescents to embark on physical activity lifestyle changes has become a key focus of many health professionals [16]. Regular physical activity can influence children’s spiritual satisfaction and enjoyment [17,18] and exercise activities which are exciting and arousing curiosity tend to attract more children participation [18]. Given that children between 6 to 12 years are usually at the stage of motor development and habits acquired during this period are carried along to the adulthood stage [19], it is therefore important to establish an adequate level of physical activity during childhood stage to maintain an appropriate level of physical activity during the adult stage necessary for healthy living.

A person’s attitude toward physical activity is defined as the individual’s tendency to favor or disfavor the behavior of physical activity [20,21]. Previous studies [2,16,18] reported that children’s attitudes toward physical activity were associated with their appreciation of how regular physical activity contribute to their personal satisfaction and fulfilment. The development of valid and reliable scales to measure children’s attitudes toward physical activity is therefore of vital importance [16]. One such important measure is the Attitude toward Physical Activity Scale (APAS). The APAS was developed in experimental studies to estimate the effect of Brain Breaks video exercise conveyed lessons [16]. It contains a demographic part and seven factors, reflecting children’s attitudes and perceptions concerning numerous perspectives of participation in physical activity, especially physical activity using video games [22].

The APAS was created to assess children’s attitudes, beliefs and self-efficacy toward physical activity [22]. The original APAS was presented in English and was shown to have adequate psychometric properties using the Rasch analysis, which found APAS to have strong Rasch reliability, person separation and gender invariance [22]. The APAS scale reliability has since been tested as part of experimental studies in several countries including Lithuania [23], Poland [18], Turkey [24], Macedonia [11] and Malaysia [25]. These previous studies have provided empirical support for the APAS scale’s reliability and validity. The purpose of this study was to determine the reliability and construct validity of the translated Chinese version of the Attitude toward Physical Activity Scale (APAS-C) for Chinese-speaking primary students.

## 2. Materials and Methods

### 2.1. Participants

The present study comprised 692 students (boy 52.6%, *n* = 364, girl 47.4%, *n* = 328) in grade 3 (*n* = 236), grade 4 (*n* = 227) and grade 5 (*n* = 229) from two schools (Name removed for confidentiality) in China. The schools were recruited based on random sampling (www.randomization.com, accessed on 18 June 2019) and received the approval from the principle of the schools. The students from all the schools participated voluntarily and with informed consent from their parents/guardians. The initial sample was estimated as 900 and twenty percent (20%) of the study participant dropped out from the study, resulting in a total of 720 students at the end of the study. However, 18 participants did not finish the survey questionnaire and another 10 failed to obtain parental consent for the study. These 28 students were excluded from the study. All the 692 participants reported themselves to be Chinese and had engaged in at least 30 min of physical activities in the last seven days.

### 2.2. Questionnaire Translation

In the present study, the original English version of the APAS scale was translated to the Chinese language using the following steps. First, a bilingual researcher translated the APAS scale from English to Chinese after considering the cultural appropriateness and content meaning of all the items. This researcher has good knowledge of the APAS scale. Second, the translated Chinese version was translated back to English by a native bilingual translator. Third, a panel of five professionals in the fields of health psychology and sports sciences, who were Chinese bilingual speakers and with over 10 years of working experience in their respective areas of expertise, further compared the original and back-translated versions of the questionnaire in terms of fidelity to the original meaning of the questionnaire items, as well as age and cultural appropriateness for Chinese children between grades 3 and 5. All differences were resolved and amended appropriately. Finally, 20 primary school students between grades 3 and 5 were invited to evaluate each item of the translated APAS-C in terms of ease of interpretation and presentation. Their responses showed that the final Chinese version (APAS-C) could be understood well by primary school students and required no further amendment. The APAS-C is available upon request from the first author.

### 2.3. Data Collection

This study was performed using a cross-sectional research design. The research methodology was assessed and endorsed by the (Name removed for confidentiality) University’s research review board and conducted in accordance with the Declaration of Helsinki. Data were collected from September 2019 to January 2020. All the participants volunteered for this study and were informed that they could withdraw at any time. Furthermore, at the beginning of the study, the participants provided their informed consent along with their parent’s permission and the approval from the school’s principal. Before beginning the data collection, trained research assistants visited each school and briefed administrators and faculty on the study procedure. The APAS-C was estimated to take between 10 to 20 min to complete.

### 2.4. Instrument

The APAS is a self-report measure consisting of 57 Likert-type items organized into seven subscales and their respective items related to physical activity behavior: (F1) Perceived benefits: promoting good health (10 items); (F2) Exercise activities importance (5 items); (F3) Learning: Video exercises promoting self-efficacy (11 items); (F4) Self-efficacy: Self-efficacy promoting video exercises used (4 items); (F5) Fun: Exercise activities that promotes motivation and amusement (14 items); (F6) Fitness: Perceived self-reliance in physical health (8 items) and (F7) Personal Best: The ability in doing your utmost best independently (5 items). Each item was rated by the participants using a four-point Likert scale ranging between strongly disagree and strongly agree. Previous studies reported the Cronbach’s alpha coefficient ranging between 0.65 and 0.76 [11] and 0.81 to 0.93 [17] for all the subscales.

### 2.5. Statistical Analysis

Data analysis was conducted using Mplus 8 [26]. Data were screened for missing values and outliers prior to the analysis. Multivariate normality assumption was checked using Mardia multivariate skewness and kurtosis tests. For the present data, the Mardia’s tests *p*-values were less than 0.05. Therefore, assumption of multivariate normality was not met. The MLR estimator (known as the Yuan–Bentler test statistic) [26], was used in the confirmatory factor analysis (CFA) as it is robust to non-normality data [26].

In line with the design of the original APAS, the 57-item APAS-C was tested with an initial hypothesized measurement model consisted of seven latent variables (subscales of APAS-C). The value of 0.40 or larger was used as criterion for acceptable standardized factor loading. Model fit was assessed again after removing a problematic item [27,28]. Further, modification indices were examined to guide decision on whether to add correlation on the items’ residuals. The goodness of fit of the measurement model was gauged by examining the values of several goodness of fit indices against their cutoff values as found in the literature. The goodness of fit indices and cutoffs used here are comparative fit index (CFI > 0.90), Tucker and Lewis index (TLI > 0.90), root mean square error of approximation (RMSEA < 0.07), the associated p-value of RMSEA (>0.05) and the standardized root mean square (SRMR < 0.08) [29].

After establishing acceptable model fit, reliability (Cronbach’s alpha) was computed for each of the seven scales. In addition, the composite reliability (CR) based on Raykov’s method [30] was computed and a value of at least 0.60 was used as cutoff for CR [31]. Further, a correlation coefficient of less than or equal to 0.85 among the factors in the final measurement model was used as cutoff for discriminant validity of the measurement model [32].

## 3. Results

### Measurement Model APAS-C

Table 1 and Table 2 displayed the results of all the tested measurement model. The APAS-C measurement model hypothesized with seven factors and 57 items (Model 1), resulted in a poor fit with the data observed based on the several fit indices cutoff values (see Model 1, Table 1). The items standardized factor loadings of the measurement Model 1 are presented in Table 2.

The analysis took several iterative rounds of CFA model re-specification and re-analysis. At each round, factor loadings and values of fit indices were examined and the CFA models was re-specified and re-analyzed. Items with standardized factor loadings below 0.40 were removed in the re-specification. The model was improved (see Table 1, Model 2); however, the fit indices for CFI and TLI were still not within the acceptable range. Modification of model also included adding error residual covariances between items within the same factor. After three rounds, the CFA model was substantially improved and all fit indices were within the acceptable range. Adding the covariance between these items’ residuals seems reasonable as the items were referred to the similar latent variable (factor). The Cronbach’s alpha for the subscales ranged from 0.50 to 0.76. The composite reliability based on the CFA final model ranged from 0.50 to 0.75. With the exceptions of the Importance Scale and the Personal Best Scale, reliabilities of all scales were above the acceptable value of 0.60 (Table 2).

The standardized factor correlation based on CFA final model ranged from 0.01 to 0.77 (see Table 3). That is, the correlation coefficients among factors were below 0.85, thus giving evidence to discriminant validity. A total of 35 items were retained in the final model and the original seven factor structure of APAS was confirmed.

## 4. Discussion

Previous studies have demonstrated benefits in academic performance when students regularly perform physical activity [24,25]. Further, the present literature’s engagement in classroom physical exercise activities contributes to enhancing students’ academic achievement and wellbeing [33,34,35]. The authors in the present study translated the APAS from English to Chinese and then tested the construct validity of the Chinese version (APAS-C) among primary school students using confirmatory factor analysis. Reliability (internal consistency) of the subscales of APAS-C was tested based on Cronbach’s alpha. Findings of the current study provided empirical evidence in support of adequate psychometric properties of APAS-C for use with Chinese primary students in grades 3 to 5. The final APAS-C fit the data well after deleting some problematic items and adding some correlated residual covariances.

The final APAS-C verified in this study was shown sufficient internal consistency with the Cronbach’s alpha ranging between and 0.50 and 0.76. A previous study reported Cronbach’s alpha coefficient of 0.65 to 0.76 [16]. In addition, all the correlations between the factors of the APAS-C were less than the cut-off value of 0.85. These results revealed that the seven factors in the final APAS-C model are unique and each factor explains a different variance than the other factor [36]. These revealed that the APAS-C has similar factor structure with the original English APAS [22].

In the study reported here, CFA was undertaken to examine and substantiate the factor structure of APAS-C. A seven-factor model was specified for the 57 items in the initial model (Model 1). This initial model was rejected based on unsatisfactory fit indices and the model was re-specified (Model 2) by deleting 19 items. This second model was again rejected because of unsatisfactory fit indices. The model was re-specified (Model 3) by adding seven correlated error residuals and removing additional three items. The final APAS-C model (Model 3) with 35 items reached adequate fit with the data. Of the total 22 deleted items, 6 were for factor Benefits, 1 for Importance, 3 for Learning, 10 for Fun, 1 for Fitness and 1 for Personal best. A total of 22 items deleted were removed because of low factor loading on their respective factors and or affect the fit indices of the model. After appropriate consultation with the expert in sport psychology and physical education, the researchers concluded that removing those items will not affect the theoretical framework of the APAS-C.

Comparing the 35 items and seven factors APAS-C in the present study with the previous APAS scale that adopted the original English version 57 item-seven factor APAS [22]. The Macedonian APAS yielded 55 items and seven factors, with Cronbach’s alpha ranging between 0.74–0.91 [11]. The Polish version yielded 57 items and six factors with Cronbach’s alpha coefficient ranging between 0.53–0.95 [18]. The Turkish version of APAS yielded 51 items and 6 factors with Cronbach’s alpha coefficient ranging between 0.71–0.93 [24]. These differences in the number of items and factors across different counties reported by different researchers may have arisen because of differences in cultures, which will lead differences in the interpretation of the items and their cultural suitability to that population.

Four correlated error residuals were added for the Learning factor, namely, (1) V3g (I learned about composition through video exercise) with V3f (I learned about writing through video exercise), (2) V3k (I learned about environmental protection from video exercise) with V3j (I learned about hygiene from video exercise), (3) V3c (I learned about art through video exercise) with V3b (I know how to do physical activity if there is a video exercise to follow) and (4) V3g (I learned about composition through video exercise) with V3e (I learned about language through video exercise). Two correlated error residuals were added for Self-confidence on physical fitness factor, namely, (1) V6g (I am confident with my hand-eye coordination) with V6f (I am confident with my rhythm) and (2) V6g (I am confident with my hand-eye coordination) with V6a (I am confident with my strength). One correlated error residual was added for the Self-efficacy factor, namely, V4d (I know which is my favorite physical activity in video exercise) with V4b (I know how to do physical activity if there is a video exercise to follow). Thus, the residual covariances indicate the assumption that the paired items have some common variances not specified in the model [37]. Additionally, these residual covariances can be included in the model when they make theoretical meaning [38,39].

Several limitations of this study are highlighted. First, the study was carried out using a cross-sectional survey, hence, generalization of the study findings must be made with caution. Secondly, the use of the self-reported measure may lead to response bias and consequently reduce the accuracy of the data obtained. However, the students were guaranteed their confidentiality and advised to answer all the items honestly. Thirdly, two of the factors (importance factor and personal best factor) have low reliability of less than 0.6 benchmarks [31] Nonetheless, the remaining five factors have reliability above 0.6 based on composite reliability and Cronbach’s alpha coefficients. Future studies should re-assess the replicability of the APAS-C for Chinese people of different ages, education levels, walks of life and health conditions.

## 5. Conclusions

In this study, the APAS-C demonstrated sufficient psychometric properties for evaluating children’s attitude toward physical activity in China. A total of 35 items remained in the final measurement model with the recommended standardized factor loadings on their respective factors. The final results are beneficial to researchers, professionals and policymakers in assessing and promoting attitude towards physical activity amongst young children.

## Figures and Tables

**Table 1:** Goodness of fit indices of the tested measurement models.

Path Models	RMSEA (90% CI)	RMSEA *p*-Value	CFI	TLI	SRMR
Model 1	0.043 (0.041, 0.045)	1.000	0.669	0.652	0.059
Model 2 ^a^	0.034 (0.030, 0.037)	1.000	0.875	0.862	0.043
Model 3 ^b^	0.029 (0.025, 0.032)	1.000	0.912	0.901	0.041

^a^ Measurement model with items deleted (v1a, v1c, v1f, v1g, v1h, v1i, v2a, v3a, v3h, v3i, v5e, v5h, v5i, v5j, v5l, v5m, v5n, v6e, v7c). ^b^ Measurement model with correlation between items’ residuals within same factor (v3g with v3f, v3k with v3j, v3c with v3b, v6g with v6f, v4d with v4b, v6g with v6a, v3g with v3e) and removal of items v5d, v5g, v5k; RMSEA = root mean square error of approximation; CFI = comparative fit indices; TLI = Tucker and Lewis index; SRMR = standardized root mean square.

**Table 2:** Standardized factor loadings for Model 1, Model 2 and Model 3 of the APAS-C.

Factors and Items	Factor Loadings			Cronbach’s Alpha	Composite Reliability
	Model 1	Model 2	Model 3/ Final Model		
Benefits				0.65	0.66
v1a	0.38	-	-		
v1b	0.50	0.51	0.51		
v1c	0.37				
v1d	0.54	0.64	0.64		
v1e	0.44	0.52	0.52		
v1f	0.35	-	-		
v1g	0.28	-	-		
v1h	0.24	-	-		
v1i	0.34	-	-		
v1j	0.48	0.58	0.59		
Importance				0.50	0.50
v2a	0.30	-	-		
v2b	0.36	0.38	0.40		
v2c	0.46	0.48	0.49		
v2d	0.45	0.46	0.47		
v2e	0.44	0.44	0.43		
Learning				0.76	0.75
v3a	0.39	-	-		
v3b	0.49	0.48	0.50		
v3c	0.58	0.54	0.54		
v3d	0.54	0.58	0.61		
v3e	0.58	0.61	0.60		
v3f	0.47	0.52	0.48		
v3g	0.54	0.60	0.53		
V3h	0.22	-	-		
v3i	0.39	-	-		
v3j	0.53	0.48	0.46		
v3k	0.48	0.56	0.44		
Self-efficacy				0.66	0.70
v4a	0.62	0.63	0.60		
v4b	0.61	0.60	0.66		
v4c	0.51	0.50	0.50		
v4d	0.55	0.56	0.64		
Fun				0.64	0.67
v5a	0.53	0.68	0.72		
v5b	0.50	0.59	0.62		
v5c	0.47	0.54	0.56		
v5d	0.40	0.37	-		
v5e	0.25	-	-		
v5f	0.45	0.44	0.38		
v5g	0.45	0.39	-		
v5h	0.39	-	-		
v5i	0.28	-	-		
v5j	0.20	-	-		
v5k	0.48	0.39	-		
v5l	0.39	-	-		
v5m	0.38	-	-		
v5n	0.36	-	-		
Fitness				0.70	0.70
v6a	0.52	0.54	0.57		
v6b	0.54	0.54	0.55		
v6c	0.61	0.61	0.60		
v6d	0.51	0.50	0.50		
v6e	0.25	-	-		
v6f	0.46	0.44	0.41		
v6g	0.47	0.46	0.47		
v6h	0.42	0.41	0.41		
Personal best				0.50	0.50
v7a	0.43	0.42	0.42		
v7b	0.36	0.35	0.34		
v7c	0.37	-	-		
v7	0.44	0.44	0.45		
v7e	0.50	0.51	0.52		

**Table 3:** Standardized factor correlation of the final measurement model of the APAS-C.

Variables	Benefit	Importance	Learning	Self-Efficacy	Fun	Fitness	Personal Best
1. Benefit	1	0.01	0.40	0.08	0.07	0.15	0.09
2. Importance		1	0.18	0.43	0.56	0.54	0.66
3. Learning			1	0.05	0.06	0.18	0.22
4. Self-efficacy				1	0.59	0.43	0.57
5. Fun					1	0.54	0.72
6. Fitness						1	0.77
7. Personal best							1

## Data Availability

The data is available upon request from the first author.
